# Supplementary material for: Nacα protects the larval fat body from cell death by maintaining cellular proteostasis in Drosophila
Source: Nat Commun. 2023 Sep 1;14:5328. doi: 10.1038/s41467-023-41103-1 (PMC10474126; doi:10.1038/s41467-023-41103-1)
Supplement: Supplementary file 1 — Supplementary Information [file 41467_2023_41103_MOESM1_ESM.pdf]

Supplementary Information

Nacα protects the larval fat body from cell death by maintaining cellular proteostasis in *Drosophila*

Takayuki Yamada[1], Yuto Yoshinari[2], Masayuki Tobo[2], Okiko Habara[1], and Takashi Nishimura[1,2*]

[1]Laboratory for Growth Control Signaling, RIKEN Center for Biosystems Dynamics Research (BDR), Kobe, Hyogo 650-0047, Japan
[2]Laboratory of Metabolic Regulation and Genetics, Institute for Molecular and Cellular Regulation, Gunma University, Maebashi, Gunma 371-8512, Japan

[*] Correspondence: t-nishimura@gunma-u.ac.jp

Supplementary Figs. 1-8
Source Data (uncropped blots)

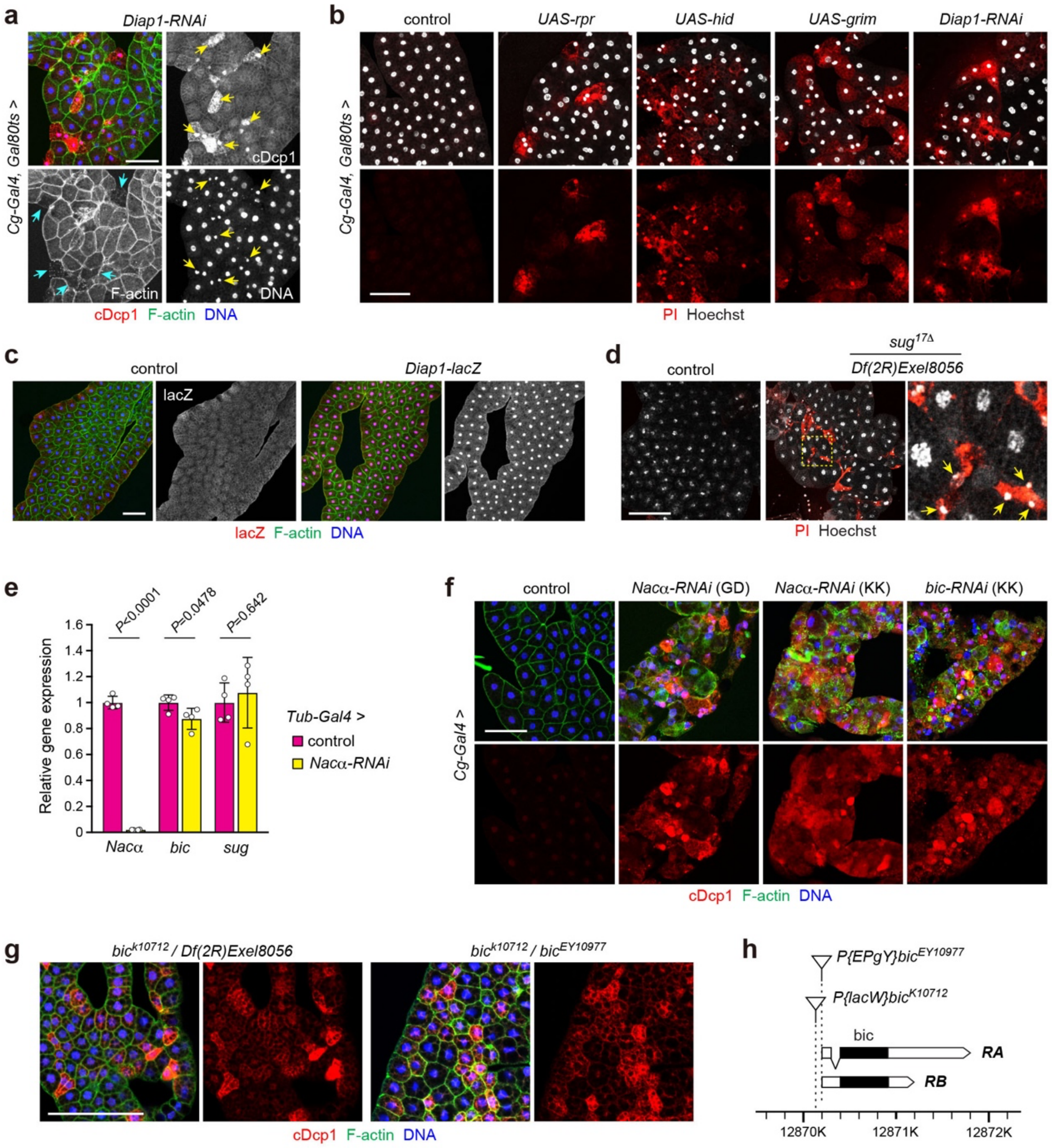

Supplementary Fig. 1: Cell death in the larval fat body, related to Figs. 1 and 2.
a Induction of fat body cell death by knockdown of the anti-apoptotic protein *Diap1*. Early-third instar larvae grown at 18 °C were incubated at 29 °C for 24 h for the transient knockdown. Fat bodies were dissected and stained for cDcp1, F-actin, and DNA. Yellow and cyan arrows indicate cDcp1-positive cells with condensed nuclei and cells with reduced cortical F-actin levels, respectively. **b** Late cell death (secondary necrosis) in the fat body as determined by PI staining. Fat bodies of the indicated genotypes were dissected from the mid-third instar (M3rd) 24 h after the temperature shift and stained with PI and Hoechst. **c** Expression pattern of *Diap1* in the larval fat body. Fat bodies were

dissected from the M3rd and stained with lacZ, F-actin, and DNA. **d** Fat body cell death as determined by PI staining. Fat bodies were dissected from the M3rd and stained with PI and Hoechst. High-magnification images of the dashed areas are shown on the right. Arrows indicate PI-positive condensed/fragmented nuclei. **e** Knockdown efficiency of *Nacα*-RNAi line (8759R-1, NIG), as determined by qRT-PCR. *Tub-Gal4* was used for the ubiquitous knockdown. Mid-second instar (M2nd) larvae were used for the analysis. Results are presented as the mean ± SD, $n = 4$ batches. Unpaired two-tailed Student's *t*-test. **f** Knockdown phenotype of *Nacα* and *bic*/*Nacβ* in the larval fat body. Fat bodies of the indicated genotypes were dissected from the M3rd and stained for cDcp1, F-actin, and DNA. GD and KK, VDRC GD and KK RNAi lines, respectively. **g** Induction of cell death in *bic* mutant fat body. Fat bodies of the indicated genotypes were dissected from the M2nd larvae and stained. **h** A schematic representation of the *bic* locus. Protein-coding regions and untranslated regions are represented by black and white boxes, respectively. The *P*-element insertion sites are marked with inverted white triangles. Scale bars, 100 μm (**a**, **b**, **c**, **d**, **f**, **g**).

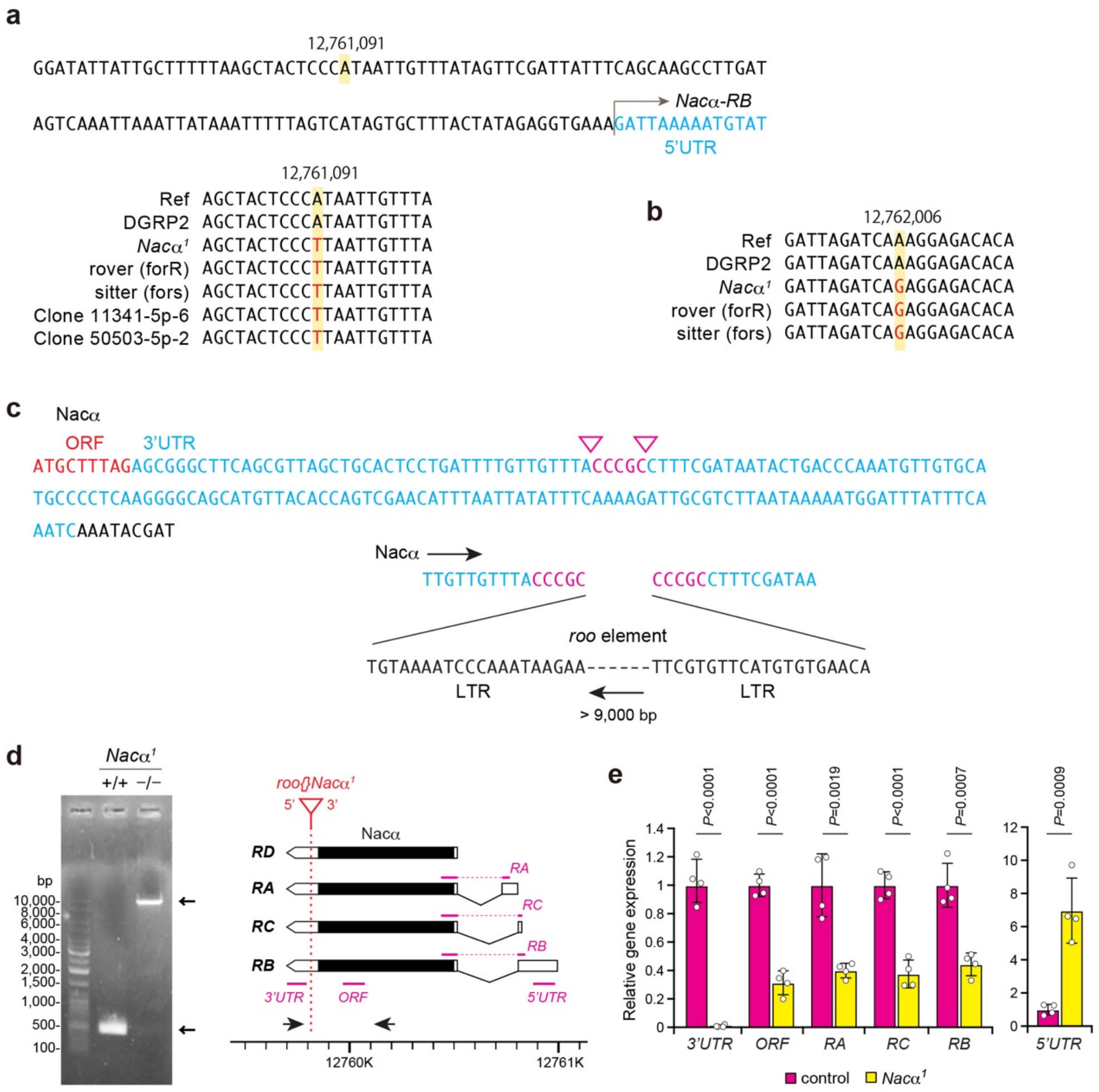

Supplementary Fig. 2: Molecular characterization of the *Nacα*^{1} mutant allele, related to Fig. 3.

a, **b** Mutations in the proximal (**a**) and distal (**b**) intergenic regions of *Nacα* gene in *Nacα*^{1} mutants. Ref, Release 6 of the *D. melanogaster* reference genome; DGRP2, *Drosophila* Genetic Reference Panel. The following sequences of *D. melanogaster* strains are shown: rover (forR), GenBank ID #CP023338.1; sitter (fors), #CP023332.1; Clone 11341-5p-6, #AB313737.1; Clone 50503-5p-2, #AB324083.1. **c** The insertion site of a *roo*-element at the *Nacα* 3'UTR as indicated by triangles. The 5 bp sequences as indicated by magenta are duplicated at the end of the LTR of the *roo* element. **d** Analysis of PCR products by agarose gel electrophoresis. A schematic representation of the *Nacα* locus is shown on the right. Protein-coding regions and untranslated regions are represented by black and

white boxes, respectively. The insertion site of the *roo*-element is marked with a red inverted triangle. Primer positions for genomic PCR analysis are indicated by black arrows. **e** Transcript levels of *Nac*α as determined by qRT-PCR. Mid-third instar larvae were used for the analysis. qPCR amplicons are indicated by purple bars in the schematic (**d**). Results are presented as the mean ± SD, $n = 4$ batches. Unpaired two-tailed Student's *t*-test.

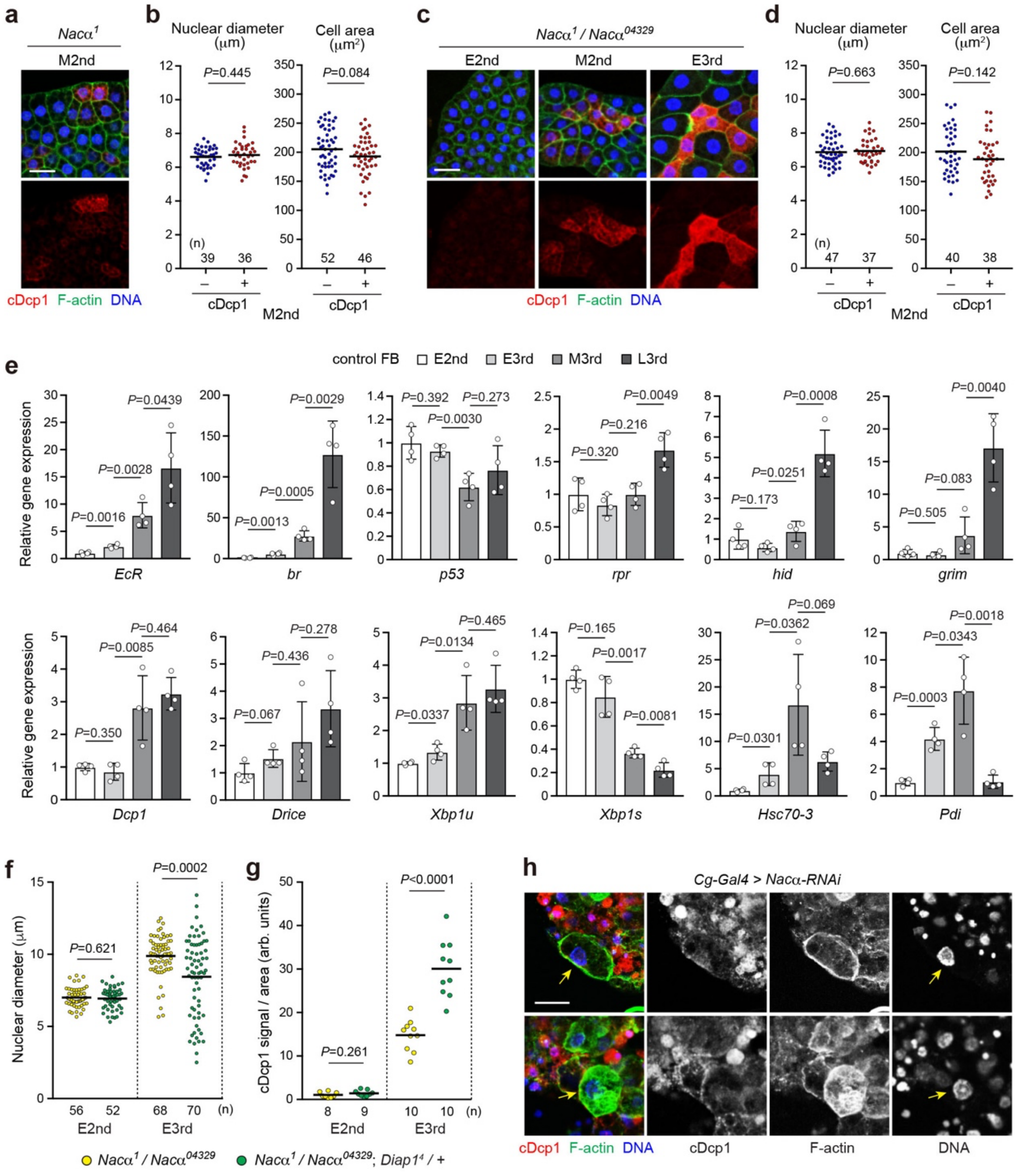

Supplementary Fig. 3: Characterization of the fat body cell death, related to Fig. 4. **a**, **b** Early phase of cell death in the *Nacα*[1] homozygous mutant fat body. Fat bodies were dissected from the mid-second instar (M2nd) and stained for cDcp1, F-actin, and DNA (**a**). Quantification of the nuclear diameter and cell area in cDcp1-negative (–) or -positive (+) fat body cells (**b**). **c**, **d** Cell death onset of the fat body in *Nacα*[1] over *Nacα*[04329] transheterozygotes. Fat bodies were dissected at each stage and stained for cDcp1, F-actin, and DNA (**c**). E2nd, early second instar; E3rd, early third instar. Quantification of the nuclear diameter and cell area in cDcp1-negative (–) or -positive (+)

fat body cells at M2nd (**d**). **e** Developmental changes in gene expression levels in the control fat body, related to **Fig. 4g**. Fat bodies (FB) of the control larvae were dissected from the indicated stages and subjected to qRT-PCR analysis. It is of note that anti-apoptotic/pro-apoptotic genes were up-regulated in the late third instar (L3rd), most likely by ecdysone signaling. Since the up-regulation of *EcR* and *br*, targets of ecdysone signaling, is already detectable in the E3rd, anti-apoptotic/pro-apoptotic genes appear to respond to high ecdysteroid titers in the L3rd. **f**, **g** Quantification of the nuclear diameter (**f**) and mean cDcp1 fluorescence intensity per tissue area (**g**), as shown in **Fig. 4h**. **h** cDcp1-negative larger cells with enlarged nuclei, as indicated by arrows. *Nacα* knockdown fat bodies were dissected from the late third instar and stained for cDcp1, F-actin, and DNA. Two examples are shown. Horizontal lines indicate the means of individual groups (**b**, **d**, **f**, **g**). Values of n indicate the number of cells (**b**, **d**), nuclei (**f**), or tissue area (**g**) from multiple animals. For appropriate panels, results are presented as the mean ± SD, $n = 4$ batches (**e**); unpaired two-tailed Welch's t-test (**b**, **d**, **f**, **g**), unpaired two-tailed Student's t-test (**e**). Scale bars, 20 µm (**a**, **c**) or 50 µm (**e**).

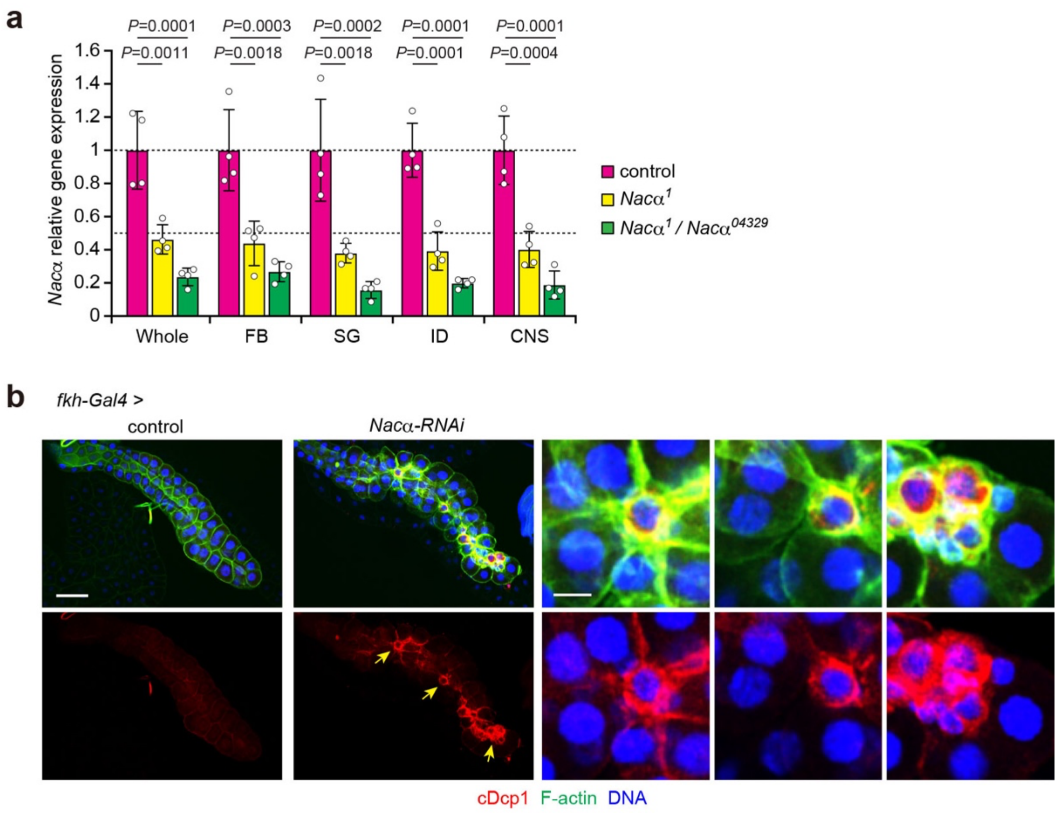

Supplementary Fig. 4: Tissue specificity of the *Nacα* mutant phenotype, related to Fig. 5.

a Relative gene expression levels of *Nacα*. Each tissue of the indicated genotypes was dissected from the mid-third instar and analyzed by qRT-PCR. Dashed lines indicate values of 1.0 and 0.5 in control larvae. Whole, whole larvae; FB, fat body; SG, salivary glands; ID, imaginal discs; CNS, central nervous system. Results are presented as the mean ± SD, $n = 4$ batches; one-way ANOVA with Dunnett's *post hoc* test. **b** Cell death in the salivary glands. Salivary glands were dissected from the mid-third instar and stained for cDcp1, F-actin, and DNA. *fkh-Gal4* was used for specific knockdown in the salivary glands. High-magnification images of cDcp1-positive cells (arrows) are shown on the right. Scale bars, 100 µm or 20 µm (right, high-magnification images).

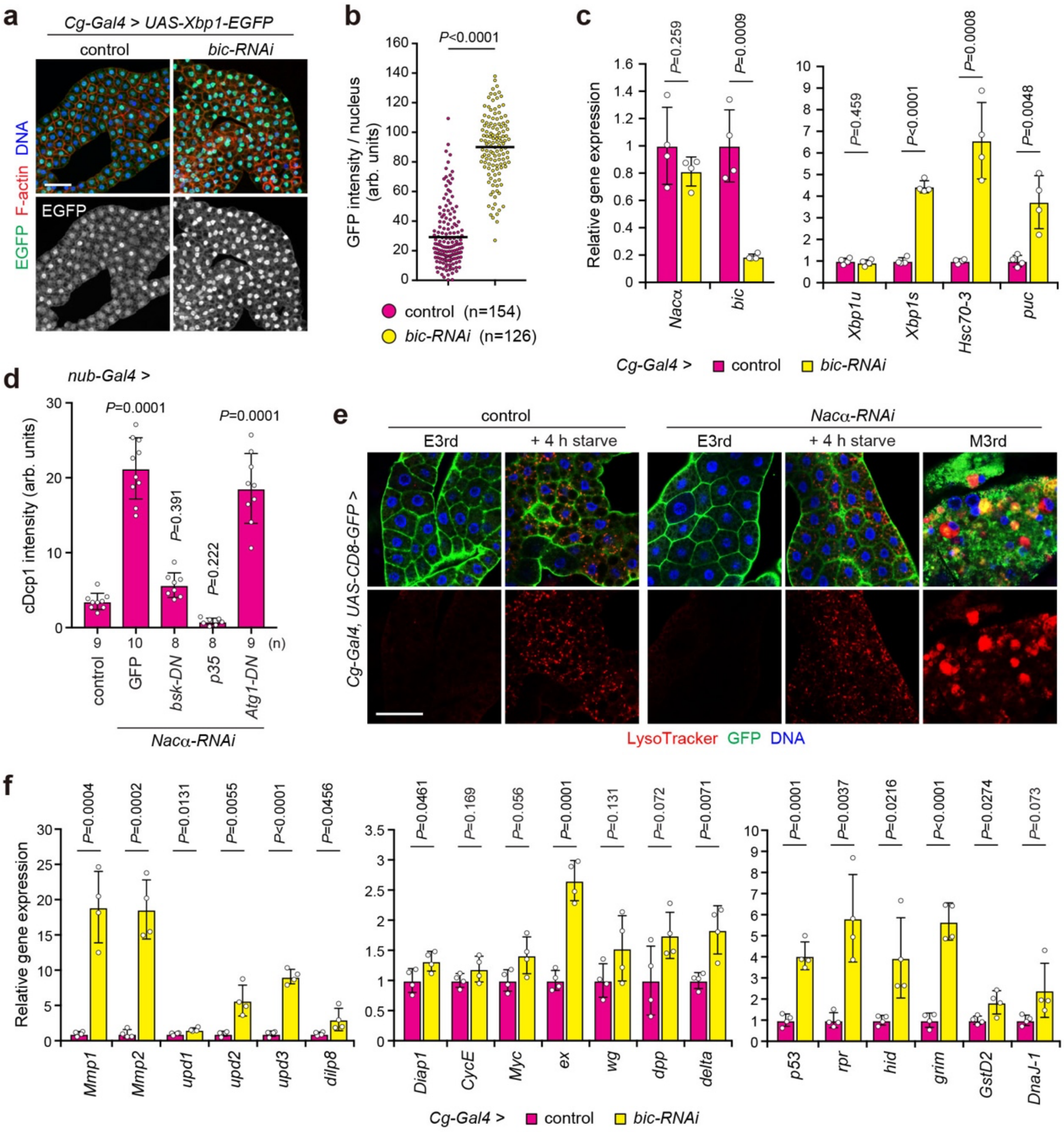

Supplementary Fig. 5: *Nacα* and *bic* knockdown phenotype in the fat body and wing discs, related to Fig. 6.

a, **b** ER stress as determined by the expression of Xbp1-GFP. Fat bodies of the indicated genotypes were dissected from the early third instar (E3rd) and stained for GFP, F-actin, and DNA (**a**). Quantification of the mean GFP fluorescence intensity per nucleus (**b**). **c** Relative gene expression levels in *bic* knockdown fat body cells. Fat bodies were dissected from the E3rd and used for qRT-PCR analysis. **d** Quantification of mean cDcp1 fluorescence intensity per wing pouch, as shown in **Fig. 6f**. **e** Autophagy in *Nacα* knockdown fat body as determined by LysoTracker staining. Fat bodies of the indicated stages and conditions were dissected and stained with LysoTracker and DNA. Membrane-

targeted CD8-GFP was used to visualize the cell morphology. M3rd, mid-third instar. *Nacα* knockdown fat body did not induce autophagy at E3rd, whereas short-term starvation (+ 4 h starve) induced LysoTracker-positive autolysosomes. *Nacα* knockdown caused a massive accumulation of autolysosomes and mislocalization of CD8-GFP at the M3rd. **f** Relative gene expression levels in *bic* knockdown fat body cells. Fat bodies were dissected from the E3rd and used for qRT-PCR analysis. Horizontal lines indicate the means of individual groups (**b**). Values of *n* indicate the number of cells (**b**) or tissues (**d**) from multiple animals. For appropriate panels, results are presented as the mean ± SD (**c**, **d**, **f**). *n* = 4 (**c**, **f**); unpaired two-sided Mann–Whitney U-test (**b**), unpaired two-tailed Student's *t*-test (**c**, **f**), One-way ANOVA with Dunnett's *post hoc* test (**d**). Scale bar, 50 µm (**a**, **e**).

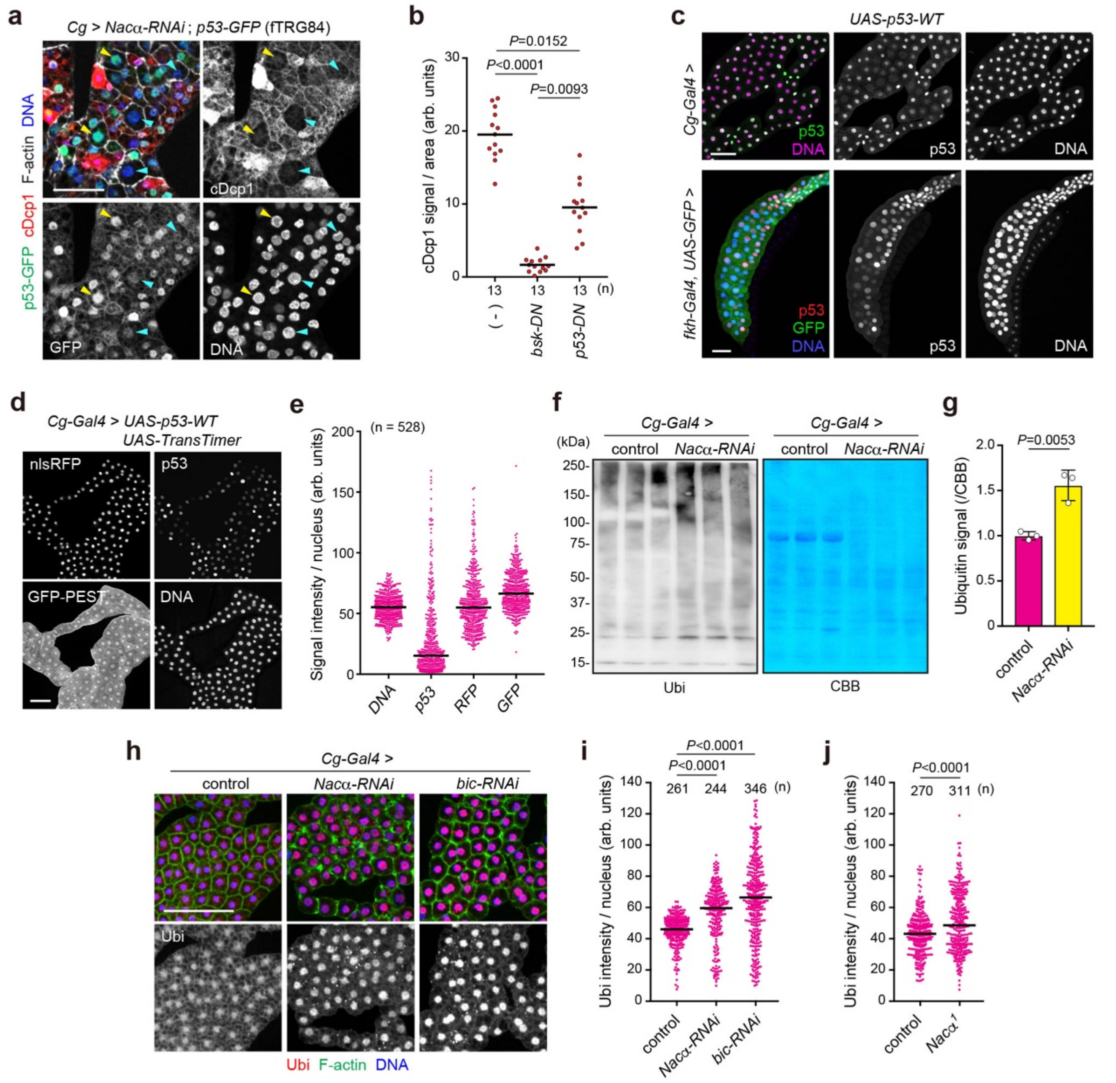

Supplementary Fig. 6: p53 and ubiquitinated protein levels in *Nacα* and *bic* mutants, related to Fig. 7.

a p53 protein levels in *Nacα* knockdown fat bodies. Fat bodies of the indicated genotypes were dissected from the mid-third instar (M3rd) and stained for GFP, cDcp1, F-actin, and DNA. Yellow and cyan arrowheads indicate p53-positive/cDcp1-negative and p53/cDcp1 double negative cells, respectively. **b** Quantification of the mean cDcp1 fluorescence intensity per tissue area, as shown in **Fig. 7d**. **c** Variable amounts of overexpressed p53 protein in the fat body and salivary gland cells. Tissues of the indicated genotypes were dissected from the M3rd and stained for p53, GFP, and DNA. **d**, **e** Variable amounts of overexpressed p53 protein in the fat body as revealed using the dual-color fluorescent reporter, TransTimer. Fat bodies were dissected from the M3rd and stained for p53, GFP, and DNA (**d**). Quantification of the mean fluorescence intensity per nucleus (**e**). **f**, **g**

Increases in ubiquitinated proteins in *Nacα* knockdown fat bodies. Fat bodies were dissected from the M3rd and used for SDS-PAGE followed by WB analysis with anti-ubiquitin antibodies (**f**, left panel). The right panel shows CBB staining of the membrane (**f**). It is of note that well-defined bands at 80kDa by CBB staining in control samples are the larval serum proteins (Lsp1/2), which increase considerably in the fat body during the third instar in response to ecdysteroids. Absence of these bands in *Nacα* knockdown fat bodies suggests that *Nacα* mutants fail to respond properly to ecdysteroids or fail to synthesize these secreted proteins due to the functional impairment of the ER. Quantification of the ubiquitin signals normalized to total protein (**g**). **h**, **i** Increases in ubiquitinated proteins in *Nacα* and *bic* knockdown fat bodies. Fat bodies of the indicated genotypes were dissected from the early third instar and stained for ubiquitin, F-actin, and DNA (**h**). Quantification of the mean ubiquitin fluorescence intensity per nucleus (**i**). **j** Quantification of ubiquitin mean fluorescence intensity per nucleus, as shown in **Fig. 7h**. Horizontal lines indicate the means (**b**) or medians (**e**, **i**) of individual groups. Values of *n* indicate the number of tissue area (**b**) or cells (**e**, **i**, **j**) from multiple animals. For appropriate panels, results are presented as the mean ± SD, $n = 3$ batches (**g**); Kruskal-Wallis test followed by Dunn's *post hoc* test (**b**, **i**), unpaired two-tailed Student's *t*-test (**g**), unpaired two-sided Mann–Whitney U-test (**j**). Scale bars, 100 µm (**a**, **c**, **d**, **h**).

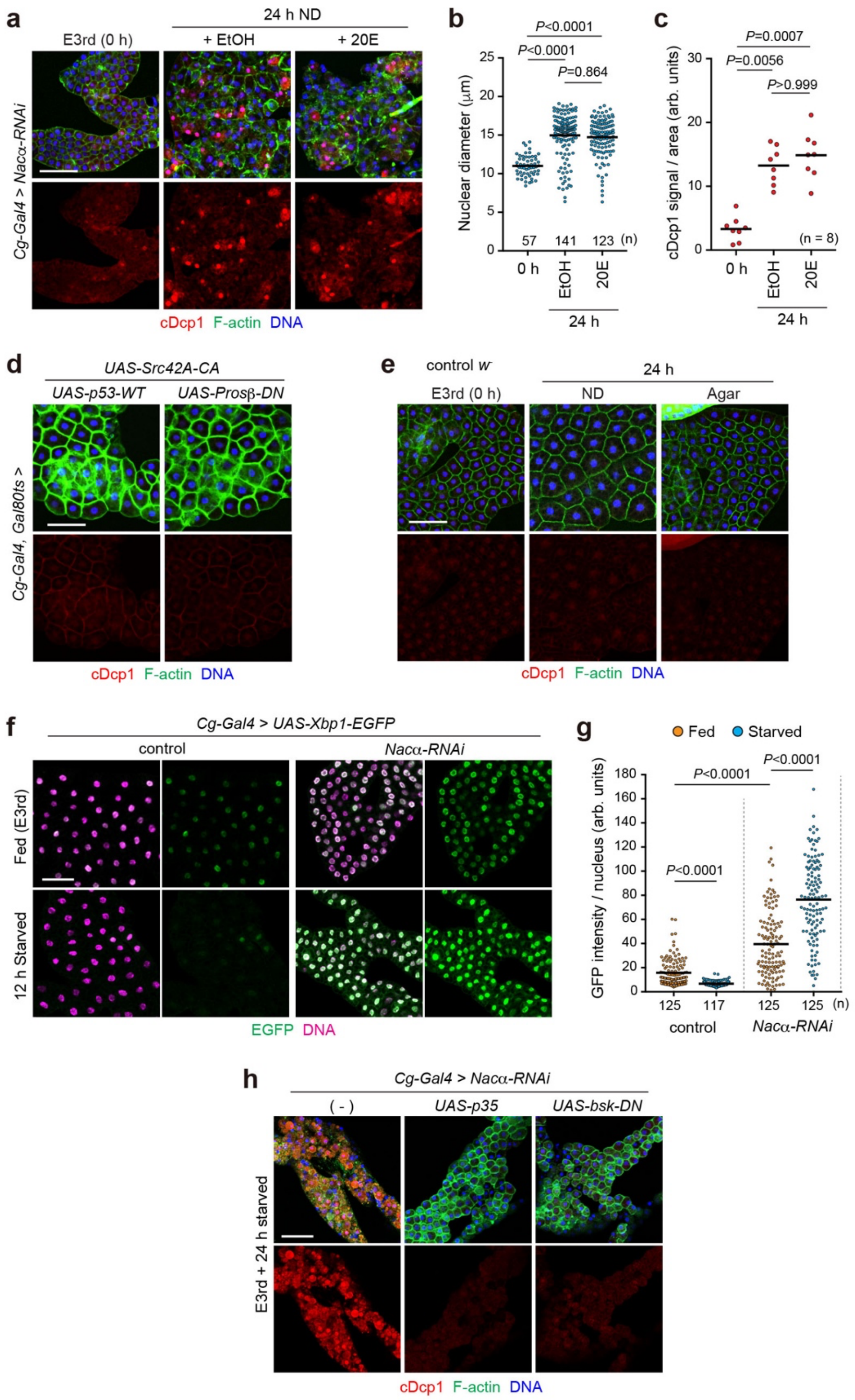

Supplementary Fig. 7: The effects of cellular and starvation stresses on the fat body, related to Fig. 8.

a, **b**, **c** The effect of ecdysteroids on the fat body cell death in *Nacα* knockdown larvae. Early third instar larvae (E3rd) were reared on a normal diet (ND) containing ethanol

(EtOH) or 20-hydroxyecdysone (20E) for 24 h. Fat bodies were dissected at the indicated time points and stained for cDcp1, F-actin, and DNA (**a**). Quantification of the nuclear diameter (**b**) and mean cDcp1 fluorescence intensity per tissue area (**c**). **d** Evaluation of the cell death phenotype in fat body. Fat bodies of the indicated genotypes were dissected from the mid-third instar (M3rd) 24 h after the temperature shift and stained for cDcp1, F-actin, and DNA. **e** Starvation does not induce cell death in the larval fat body. Control E3rd larvae were reared on an ND or fasted on an agar-only diet (agar) for 24 h. Fat bodies were dissected and stained for cDcp1, F-actin, and DNA. **f**, **g** ER stress under fed and fasted conditions as determined by the expression of Xbp1-GFP. Fat bodies of the indicated genotypes were dissected and stained for GFP and DNA (**f**). Quantification of the mean GFP fluorescence intensity per nucleus (**g**). **h** Starvation-induced cell death in *Nac*α knockdown fat body depends on JNK and caspase. Fat bodies of the indicated genotypes were dissected and stained for cDcp1, F-actin, and DNA. Horizontal lines indicate the means of individual groups (**b**, **c**, **g**). Values of *n* indicate the number of nuclei (**b**, **g**) or tissue area (**c**) from multiple animals. Kruskal-Wallis test followed by Dunn's *post hoc* test (**b**, **c**, **g**). Scale bars, 100 μm (**a**, **d**, **e**, **h**), or 50 μm (**f**).

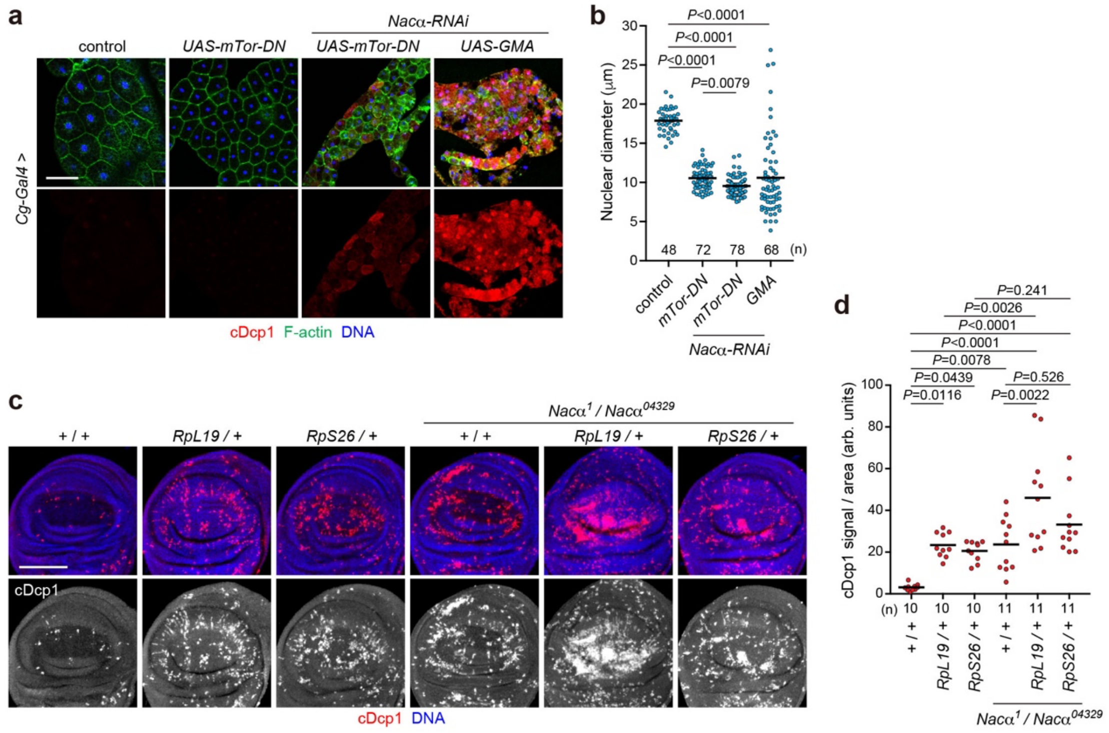

Supplementary Fig. 8: The effects of reducing protein synthesis and proteotoxic stress on the fat body and wing discs, related to Fig. 9.

a, **b** Evaluation of cell death and nuclear size in the fat body. Fat bodies of the indicated genotypes were dissected from the late third instar (L3rd) and stained for cDcp1, F-actin, and DNA (**a**). *UAS-GMA*, GFP-tagged actin-binding domain of *Drosophila* moesin, was used as a control. Quantification of the nuclear diameter (**b**). **c**, **d** Apoptosis in wing discs. Wing discs of the indicated genotypes were dissected from the L3rd and stained for cDcp1 and DNA (**c**). Quantification of the mean cDcp1 fluorescence intensity per tissue area (**d**). Horizontal lines indicate the means of individual groups (**b**, **d**). Values of *n* indicate the number of nuclei (**b**) or tissue area (**d**) from multiple animals. Kruskal-Wallis test followed by Dunn's *post hoc* test (**b**), One-way ANOVA with Tukey's *post hoc* test (**d**). Scale bars, 100 μm (**a**, **c**).

Fig. 7a

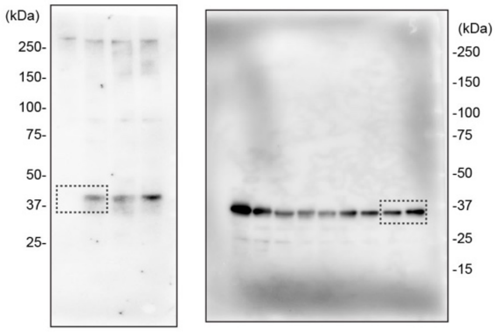

Supplementary Fig. 6f

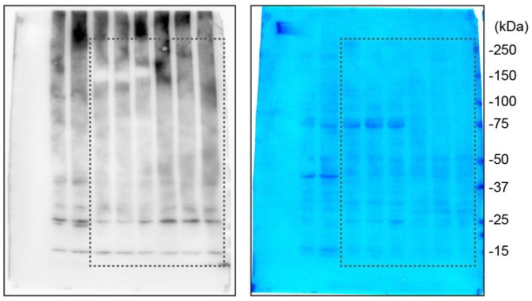

Source Data (uncropped blots)
