# Supplementary material for: Nacα protects the larval fat body from cell death by maintaining cellular proteostasis in Drosophila
Source: Nat Commun. 2023 Sep 1;14:5328. doi: 10.1038/s41467-023-41103-1 (PMC10474126; doi:10.1038/s41467-023-41103-1)
Supplement: Supplementary file 3 — Description of Additional Supplementary Files [file 41467_2023_41103_MOESM3_ESM.pdf]

Description of Additional Supplementary Files

File Name: Supplementary Data 1

Description: Summary of SNPs and indels within the narrowed down region (FlyBase region 12,743,139–12,780,370) in *Nac*□^{1} mutants. Grayed rows indicate the position of each gene.

File Name: Supplementary Data 2

Description: List of oligonucleotides used for qRT-PCR analyses.
