# Supplementary material for: Nacα protects the larval fat body from cell death by maintaining cellular proteostasis in Drosophila
Source: Nat Commun. 2023 Sep 1;14:5328. doi: 10.1038/s41467-023-41103-1 (PMC10474126; doi:10.1038/s41467-023-41103-1)
Supplement: Supplementary file 6 — Reporting Summary [file 41467_2023_41103_MOESM6_ESM.pdf]

nature portfolio

Corresponding author(s): Takashi Nishimura

Last updated by author(s): Aug 2, 2023

Reporting Summary

Nature Portfolio wishes to improve the reproducibility of the work that we publish. This form provides structure for consistency and transparency in reporting. For further information on Nature Portfolio policies, see our Editorial Policies and the Editorial Policy Checklist.

Statistics

For all statistical analyses, confirm that the following items are present in the figure legend, table legend, main text, or Methods section.

n/a	Confirmed	
☐	☒	The exact sample size (*n*) for each experimental group/condition, given as a discrete number and unit of measurement
☐	☒	A statement on whether measurements were taken from distinct samples or whether the same sample was measured repeatedly
☐	☒	The statistical test(s) used AND whether they are one- or two-sided<br>*Only common tests should be described solely by name; describe more complex techniques in the Methods section.*
☒	☐	A description of all covariates tested
☐	☒	A description of any assumptions or corrections, such as tests of normality and adjustment for multiple comparisons
☐	☒	A full description of the statistical parameters including central tendency (e.g. means) or other basic estimates (e.g. regression coefficient) AND variation (e.g. standard deviation) or associated estimates of uncertainty (e.g. confidence intervals)
☐	☒	For null hypothesis testing, the test statistic (e.g. *F*, *t*, *r*) with confidence intervals, effect sizes, degrees of freedom and *P* value noted<br>*Give P values as exact values whenever suitable.*
☒	☐	For Bayesian analysis, information on the choice of priors and Markov chain Monte Carlo settings
☒	☐	For hierarchical and complex designs, identification of the appropriate level for tests and full reporting of outcomes
☒	☐	Estimates of effect sizes (e.g. Cohen's *d*, Pearson's *r*), indicating how they were calculated

Our web collection on statistics for biologists contains articles on many of the points above.

Software and code

Policy information about availability of computer code

Data collection	Zen 2009 and Zen 2.3 (Zeiss) were used for image acquisition.
Data analysis	Fiji 2.3.0 for image analysis, Microsoft Excel 16.54 and GraphPad Prism 7 for statistic analysis.

For manuscripts utilizing custom algorithms or software that are central to the research but not yet described in published literature, software must be made available to editors and reviewers. We strongly encourage code deposition in a community repository (e.g. GitHub). See the Nature Portfolio guidelines for submitting code & software for further information.

Data

Policy information about availability of data

All manuscripts must include a data availability statement. This statement should provide the following information, where applicable:

- Accession codes, unique identifiers, or web links for publicly available datasets
- A description of any restrictions on data availability
- For clinical datasets or third party data, please ensure that the statement adheres to our policy

The source data underlying all main and supplementary figures are provided as a Source Data file. Sequences of oligonucleotides used in this study are included in Supplementary Data file 2. WGS data are deposited in the SRA under accession number PRJNA941141 [https://www.ncbi.nlm.nih.gov/sra/SRP425742].

Research involving human participants, their data, or biological material

Policy information about studies with human participants or human data. See also policy information about sex, gender (identity/presentation), and sexual orientation and race, ethnicity and racism.

Reporting on sex and gender	n/a
Reporting on race, ethnicity, or other socially relevant groupings	n/a
Population characteristics	n/a
Recruitment	n/a
Ethics oversight	n/a

Note that full information on the approval of the study protocol must also be provided in the manuscript.

Field-specific reporting

Please select the one below that is the best fit for your research. If you are not sure, read the appropriate sections before making your selection.

☒ Life sciences ☐ Behavioural & social sciences ☐ Ecological, evolutionary & environmental sciences

For a reference copy of the document with all sections, see nature.com/documents/nr-reporting-summary-flat.pdf

Life sciences study design

All studies must disclose on these points even when the disclosure is negative.

Sample size	No statistical predetermination of sample size was performed. We used standard values according to the general use in the research field and determined sample size based on our previous experiments and expertise over the past years (Yamada et al., Development, 2018; Yamada et al., Development, 2019; Matsushita and Nishimura, Commun Biol, 2020; Nishimura, Curr Biol, 2020; Yamada et al., Nature Metabolism, 2020).
Data exclusions	No data were excluded from the analysis.
Replication	Experiments were replicated at least twice using independently rared populations to ensure reproducibility. Alternatively, samples were collected from independently rared populations. All attempts at replication were successful.
Randomization	Within genotypes, staged animals were allocated randomly to different dietary conditions and collected randomly. Samples were treated in a randomized manner during the analysis.
Blinding	Blinding was not applicable because the investigator who set up the experiments was the same person doing sample collection and analysis. However, each experiment was associated with proper controls, and samples were collected and analyzed under the identical conditions.

Reporting for specific materials, systems and methods

We require information from authors about some types of materials, experimental systems and methods used in many studies. Here, indicate whether each material, system or method listed is relevant to your study. If you are not sure if a list item applies to your research, read the appropriate section before selecting a response.

Materials & experimental systems

n/a	Involved in the study	
☐	☒	Antibodies
☒	☐	Eukaryotic cell lines
☒	☐	Palaeontology and archaeology
☐	☒	Animals and other organisms
☒	☐	Clinical data
☒	☐	Dual use research of concern
☒	☐	Plants

Methods

n/a	Involved in the study	
☒	☐	ChIP-seq
☒	☐	Flow cytometry
☒	☐	MRI-based neuroimaging

Antibodies

Antibodies used

rabbit anti-cleaved Caspase-3 (#9661, Cell Signaling Technology)
rabbit anti-cleaved Dcp-1 (#9578, Cell Signaling Technology)
mouse anti-Ubiquitin (P4D1, sc-8017, Santa Cruz)
mouse anti-LacZ (40-1a, Developmental Studies Hybridoma Bank)
chicken anti-GFP (ab13970, Abcam)
Alexa Fluor 488-conjugated goat anti-chicken IgY (A-11039, Thermo Fisher Scientific)
Alexa Fluor 488-conjugated goat anti-mouse IgG (A-11029, Thermo Fisher Scientific)
Alexa Fluor 555-conjugated goat anti-rabbit IgG antibodies (A-21428, Thermo Fisher Scientific)
Alexa Fluor 555-conjugated goat anti-mouse IgG (A-21424, Thermo Fisher Scientific)
monoclonal anti-p53 (C-11, sc-55476, Santa Cruz)
goat polyclonal anti-GAPDH (IMG-3073, Imgenex)
HRP-linked anti-mouse IgG (#7076, Cell Signaling Technology)
HRP-linked anti-goat IgG (ab97110, Abcam)

Validation

Specificity of the antibodies has been validated by the manufacturer for immunofluorescence analysis and Western blotting as indicated on the manufacturer's website.

rabbit anti-cleaved Caspase-3 (#9661, Cell Signaling Technology) https://www.cellsignal.com/products/primary-antibodies/cleaved-caspase-3-asp175-antibody/9661
rabbit anti-cleaved Dcp-1 (#9578, Cell Signaling Technology) https://www.cellsignal.com /products/primary-antibodies/cleaved-drosophila-dcp-1-asp215-antibody/9578
mouse anti-Ubiquitin (P4D1, sc-8017, Santa Cruz) https://www.scbt.com/p/ubiquitin-antibody-p4d1
mouse anti-LacZ (40-1a, Developmental Studies Hybridoma Bank) https://dshb.biology.uiowa.edu/40-1a
chicken anti-GFP (ab13970, Abcam) https://www.abcam.com/products/primary-antibodies/gfp-antibody-ab13970.html
Alexa Fluor 488-conjugated goat anti-chicken IgY (A-11039, Thermo Fisher Scientific) https://www.thermofisher.com/antibody/product/Goat-anti-Chicken-IgY-H-L-Secondary-Antibody-Polyclonal/A-11039
Alexa Fluor 488-conjugated goat anti-mouse IgG (A-11029, Thermo Fisher Scientific) https://www.thermofisher.com/antibody/product/Goat-anti-Mouse-IgG-H-L-Highly-Cross-Adsorbed-Secondary-Antibody-Polyclonal/A-11029
Alexa Fluor 555-conjugated goat anti-rabbit IgG antibodies (A-21428, Thermo Fisher Scientific) https://www.thermofisher.com/antibody/product/Goat-anti-Rabbit-IgG-H-L-Cross-Adsorbed-Secondary-Antibody-Polyclonal/A-21428
Alexa Fluor 555-conjugated goat anti-mouse IgG (A-21424, Thermo Fisher Scientific) https://www.thermofisher.com/antibody/product/Goat-anti-Mouse-IgG-H-L-Highly-Cross-Adsorbed-Secondary-Antibody-Polyclonal/A-21424
monoclonal anti-p53 (C-11, sc-55476, Santa Cruz) https://www.scbt.com/p/p53-antibody-c-11
goat polyclonal anti-GAPDH (IMG-3073, Imgenex) https://www.novusbio.com/products/gapdh-antibody_nb300-320
HRP-linked anti-mouse IgG (#7076, Cell Signaling Technology) https://www.cellsignal.com/products/secondary-antibodies/anti-mouse-igg-hrp-linked-antibody/7076
HRP-linked anti-goat IgG (ab97110, Abcam) https://www.abcam.com/products/secondary-antibodies/donkey-goat-igg-hl-hrp-ab97110.html

Animals and other research organisms

Policy information about studies involving animals; ARRIVE guidelines recommended for reporting animal research, and Sex and Gender in Research

Laboratory animals

The Drosophila melanogaster strains used in this study are described below. Mixed sexes at the indicated stages in the Figure legends were used for experiments.
w1118 (used as a control), sugdelta17 (a gift from Dr. Ville Hietakangas), UAS-bsk-DN, nub-Gal4 (gifts from Dr. SaKan Yoo), UAS-Atg1-DN (a gift from Dr. Thomas P. Neufeld), and UAS-grim (a gift from Dr. Michael B. O'Connor). Nacalpha-RNAi (8759R-1), bic-RNAi (3644R-1), and Diap1-RNAi (HMS00752) were obtained from the National Institute of Genetics (NIG) Drosophila Stock Center. P{lacW}RpL19k03704 (102285), Diap14 (108011), P{lacW}Diap1j5C8 (111396), and P{SUPor-P}RpS26KG00230 (114620) were obtained from the Kyoto Drosophila Genetic Resource Center (DGRC). Nacalpha-RNAi (GD, 36017), Nacalpha-RNAi (KK, 109114), bic-RNAi (KK, 104718), and p53-GFP (fTRG84, 318453) were obtained from the Vienna Drosophila RNAi Center (VDRC). The following stocks were obtained from the Bloomington Drosophila stock center (BDSC): UAS-p35 (5073), Tub-Gal4 (5138), UAS-rpr (5824), UAS-Hsc70-3-D231S (5841), UAS-hep-CA (6406), UAS-Src42A-CA (6410), UAS-p53-259H (6582), UAS-p53-WT (6584), UAS-Prosbeta61, UASProsbeta21 (6787), Cg-Gal4 (7011), UAS-mTor.TED (7013), Df(2R)Exel7123 (7870), Df(2R)Exel80565 (7916), UAS-EcRA-W650A (9451), P{lacW}bicK10712 (10998), P{PZ}Nacalpha04329 (11371), P{SUPor-P}bicKG01035 (13186), UAS-Sod2 (24494), UAS-Cat (24621), UAS-GMA (31775), UAS-Xbp1-EGFP (60730), UAS-psn+14 (63243), UAS-hid (65403), fkh-Gal4 (78061), UAS-GC3Ai (84343), and UAS-TransTimer (93411).

Wild animals

No wild animals were used in the study.

Reporting on sex

Mixed sexes at the indicated stages in the Figures and Figure legends were used for experiments.

Field-collected samples

No field collected samples were used in the study.

Ethics oversight

This study did not require an ethical approval.

Note that full information on the approval of the study protocol must also be provided in the manuscript.
